# Hydrogen Evolution Reaction of Electrodeposited Ni‐W Films in Acidic Medium and Performance Optimization Using Machine Learning

**DOI:** 10.1002/cssc.202400444

**Published:** 2024-11-13

**Authors:** Roger de Paz‐Castany, Konrad Eiler, Aliona Nicolenco, Maria Lekka, Eva García‐Lecina, Guillaume Brunin, Gian‐Marco Rignanese, David Waroquiers, Thomas Collet, Annick Hubin, Eva Pellicer

**Affiliations:** ^1^ Physics Department Universitat Autònoma de Barcelona Campus de la UAB 08193 Bellaterra, Cerdanyola del Vallès Spain; ^2^ CIDETEC Basque Research and Technology Alliance (BRTA) P° Miramón 196 20014 San Sebastián Spain; ^3^ Matgenix, A6K Advanced Engineering Centre Sq. des Martyrs 1 6000 Charleroi Belgium; ^4^ SURF Department Vrije Universiteit Brussel Pleinlaan 2 1050 Brussels Belgium

**Keywords:** Nickel-tungsten, Electrodeposition, Hydrogen evolution reaction, Sulfuric acid, Machine learning

## Abstract

Ni−W alloy films were electrodeposited from a gluconate aqueous bath at pH=5.0, at varying current densities and temperatures. While there is little to no difference in composition, i. e., all films possess ~12 at.% W, their activity at hydrogen evolution reaction (HER) in acidic medium is greatly influenced by differences in surface morphology. The kinetics of HER in 0.5 M H_2_SO_4_ indicates that the best performing film was obtained at a current density of −4.8 mA/cm^2^ and 50 °C. The Tafel slopes (*b*) and the overpotentials at a geometric current density of −10 mA/cm^2^ (*η*_10_) obtained for 200 cycles of linear sweep voltammetry (LSV) from a set of films deposited using different parameters were fed into a machine learning algorithm to predict optimum deposition conditions to minimize *b*, *η*_10_, and the degradation of samples over time. The optimum deposition conditions predicted by the machine learning model led to the electrodeposition of Ni−W films with superior performance, exhibiting *b* of 33–45 mV/dec and an *η*_10_ of 0.09–0.10 V after 200 LSVs.

## Graphical abstract

Electrodeposited Ni−W films with tunable morphology and nearly constant tungsten content (12 at.% W) show varying activity towards hydrogen evolution reaction (HER) in acidic medium. Films with superior HER activity have been predicted by a machine learning (ML) algorithm and performed experimentally. The durability of the Ni−W films in acidic medium under continued electrolysis for 7 days is demonstrated.

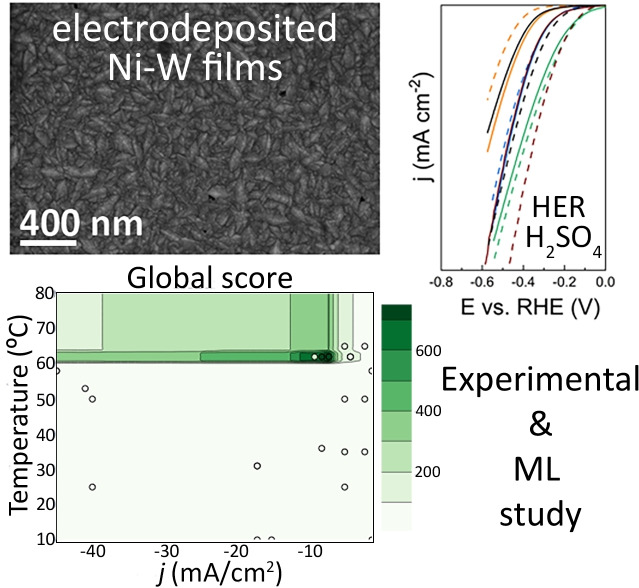

## Introduction

The need to reduce our dependence on fossil fuels with the help of green energy and the development of efficient solutions for energy storage are among the most urgent challenges of current times. In this context, hydrogen technology emerges as a key factor, offering a clean fuel with a high energy density that can be produced through water electrolysis. However, the widespread usage of hydrogen as an energy vector is hindered by a significant bottleneck, namely, most electrodes contain platinum group metals (PGM) due to their high catalytic activity and stability. The presence of PGMs increases the cost of the catalyst due to their scarcity which is expected to increase in the following years.^[1]^ This impending cost escalation makes hydrogen and fuel cell technology too costly to be implemented on a larger scale at the current state of the art. Hence, there is a need to develop alternative catalysts not reliant on PGMs. For hydrogen production in an electrolyzer, the main reaction is the hydrogen evolution reaction (HER). Depending on the electrolyzer, this reaction can take place in either acidic or alkaline media, though the acidic process is usually more efficient.

Nickel is a very good candidate for HER due to its catalytic activity, price, and abundance.[^2^, ^3^, ^4^, ^5^] When evaluating the hydrogen‐metal bond energies of various common metals, it becomes evident that Ni possesses a nearly ideal value, presenting a hydrogen bond energy slightly lower than that of PGMs.[^2^, ^3^] Tungsten is also a very promising element which in this case presents a slightly higher H−M bond energy than PGMs.^[6]^ When alloying both metals, an enhancement of the catalytic properties of Ni has been observed.[^7^, ^8^, ^9^] This is why the development of Ni−W alloys has been proposed to address the limitations associated with traditional catalysts. The solubility of W in the Ni face‐centered cubic (fcc) lattice is about 10 at.% at room temperature. At higher W concentrations (between 10 and 50 at.%), Ni_4_W may be formed.^[10]^

Many Ni alloys can be readily produced by aqueous electrodeposition, which is a rather fast and efficient process with a low waste balance. While tungsten cannot be electrochemically reduced alone, it can be co‐deposited with Ni, which acts as an inductor for the deposition of W, using tungstate as a precursor.[^11^, ^12^, ^13^] Co‐deposition of Ni−W is well documented in the literature, mostly from ammonia‐citrate electrolytes, at pH=8–9.[^14^, ^15^, ^16^, ^17^, ^18^] The research has been mainly triggered by the fact that the resulting coatings show good corrosion resistance and mechanical properties, including superior wear resistance, owing to the incorporation of a sufficient amount of tungsten in the coatings.[^17^, ^19^] Such incorporation, for amounts of ca. 50 wt.%, causes full amorphization of the coatings. Besides the inherent metallurgical interest towards the Ni−W system, electrodeposited Ni−W alloys have also demonstrated activity at HER in alkaline media.[^18^, ^23^, ^24^, ^25^, ^26^] Their HER performance in acidic medium has been comparatively less explored.[^7^, ^27^, ^28^] Rashkov *et al*. have electrodeposited Ni−W on carbon fibers and investigated the kinetics of hydrogen evolution in sulfuric acid.^[27]^ In another study, Ni−W nanoparticles have been electrodeposited and also subject to HER in acidic medium,^[7]^ though the amount of W in the nanoparticles was not provided. Additionally, Cu nanowires have been coated with a Ni−W coating by electrodeposition and the Ni−W alloy with 4 at.% W showed the highest activity among the catalysts with an overpotential of 56 mV at −10 mA cm^−2^ in 0.5 M H_2_SO_4_.^[28]^

In this work, we present a comprehensive study of the electrodeposition of Ni−W alloys from a gluconate bath at slightly acidic pH (5.0). The dependence of morphology and composition on current density and temperature during the deposition is scrutinized. As a matter of fact, gluconate has been regarded as a cheap and environmentally safe additive in nickel electrodeposition from acidic bath.^[29]^ The electrochemical performance and durability of the deposited alloy films is demonstrated for HER in acidic media (0.5 M H_2_SO_4_). Interestingly, while the W content remains rather constant, we demonstrate that the electrocatalytic activity can be largely modulated. The performances of the deposited films are further optimized thanks to a machine learning (ML) model. Indeed, in the last few years, computational methods such as ML have been used to accelerate materials design and exploration,[^30^, ^31^, ^32^, ^33^, ^34^, ^35^] as well as to optimize lab processes.^[36]^ ML is a powerful tool that can drastically reduce the amount of experimental work needed to reach optimal performance. In this work, instead of relying on an empirical trial‐and‐error approach, which is common practice for electrochemical synthesis, the electrodeposition parameters are optimized by a ML model that is trained on an initial set of already‐performed experiments. These suggestions are then realized experimentally, hence saving time and resources by limiting the number of samples to deposit. Using the so‐called active learning method, where the additional results are fed to the ML model, we reach optimal electrocatalytic activity for the deposited Ni−W films.

## Results and Discussion

### Electrodeposition of Ni‐W Films

**Figure 1:**
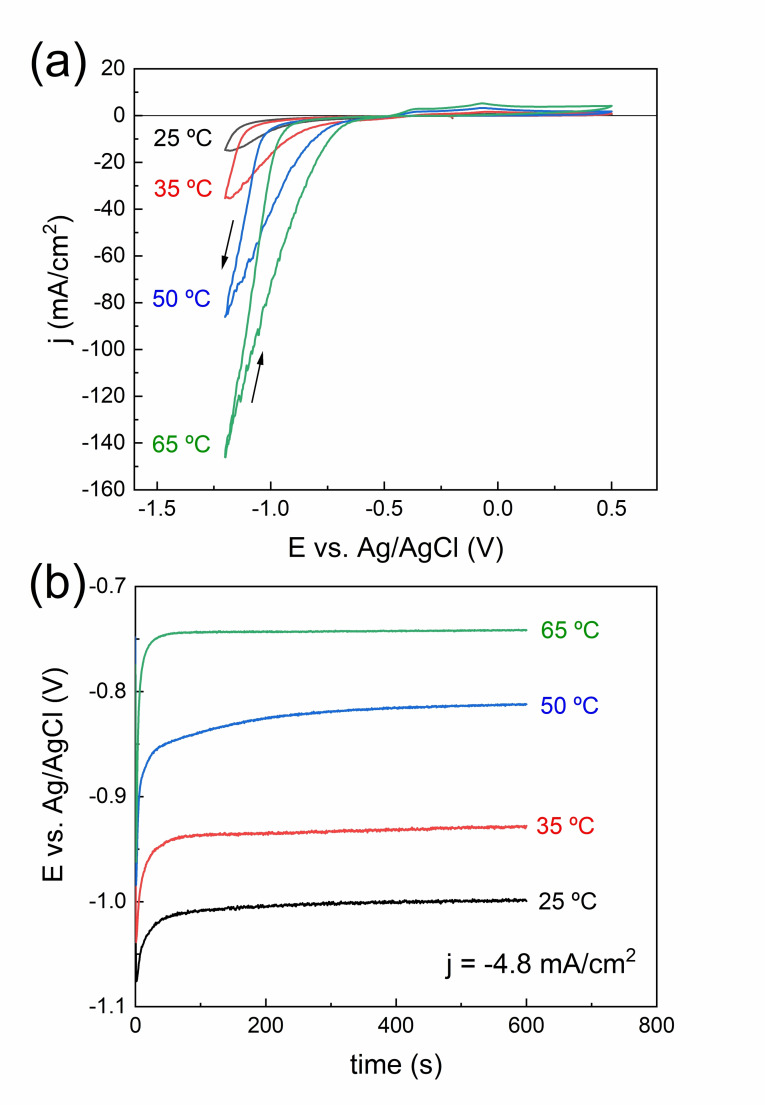
CVs recorded on Si/Ti/Au surface at 50 mV/s and at the indicated bath temperatures, under ω=100 rpm (a), galvanostatic curves for the deposition of Ni−W films at a fixed current density of −4.8 mA/cm^2^, ω=100 rpm, and varying bath temperatures (b).

Before deposition of the Ni−W films, the electrolyte (0.11 M NiSO_4_⋅7 H_2_O, 0.05 M Na_2_WO_4_⋅2 H_2_O, 0.5 M NaC_6_H_11_O_7_, 0.65 M H_3_BO_3_, pH=5.0) was characterized by cyclic voltammetry (CV). Figure 1a shows the CV curves recorded on Au‐metalized Si substrate at varying temperatures (25 °C–65 °C). The onset of deposition shifts towards more positive values as the bath temperature increases, indicating favored mass transport. Deposition of Ni−W on Au starts at around −0.8 V with a sharp increase in the current density. Notably, the anodic branch shows a comparably low oxidation current, leading to low oxidation to reduction charge ratios (Q_ox_/Q_red_), which might indicate either a low current efficiency or unfavorable coating dissolution.^[37]^ According to the CV fingerprint, a sufficiently broad window of current densities potentially leading to an appreciable Ni−W deposition, namely between −1 and −40 mA/cm^2^, was chosen for the subsequent preparation of the films.

Figure 1b shows the *E*‐t curves recorded during the electrodeposition of Ni−W films at a fixed current density of −4.8 mA/cm^2^ and varying temperature. The curves show the characteristic nucleation spike followed by a relaxation of the potential towards a stationary value, which shifts towards more positive values as temperature increases, in agreement with the displacements observed in the cathodic branch of the CVs. The *E*‐t curves recorded at other current densities are given in the Supplementary Information (Figure S1).

### Characterization of Ni‐W Films

**Figure 2:**
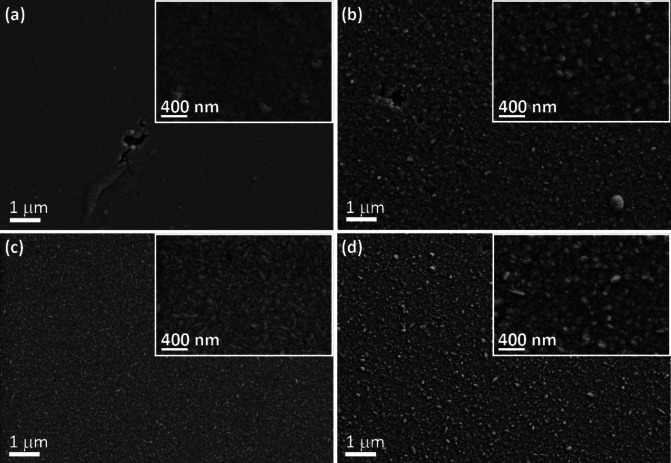
SEM images of the Ni−W films electrodeposited at a fixed current density of −4.8 mA/cm^2^ at 25 °C (a), 35 °C (b), 50 °C (c), and 65 °C (d). Insets show a magnified detail of the grain morphology.

Figure 2 shows the scanning electron microscopy (SEM) images of films electrodeposited at a fixed current density of −4.8 mA/cm^2^ but different bath temperatures. Although a few voids are observed, the films are nearly crack‐free. A significant difference in the morphology of the electrodeposited films can be observed as a function of bath temperature. At the lowest deposition temperature (25 °C), the coating consists of fine, nodular grains, leading to a rather smooth surface (Figure 2a). The grains become more elongated at 35 °C, with better resolved features (Figure 2b), and acicular‐shaped grains are clearly distinguished at 50 °C (Figure 2c). With further increase in temperature, rounded grains form, giving rise to a morphology similar to that obtained at 35 °C (Figure 2d). Energy‐dispersive X‐ray spectroscopy (EDX) was carried out to determine the amount of tungsten incorporated into the films (Table 1). The results indicate that the tungsten content remains rather constant (ca. 12 at.%) in spite of the observed changes in grain morphology.^[20]^ Similar results are obtained at applied current densities of −1 and −40 mA/cm^2^ (see Table S1). Complementary inductively coupled plasma optical emission spectroscopy (ICP‐OES) was carried out after full chemical digestion of the films. First of all, the agreement between EDX and ICP data is verified, with a discrepancy of 0.3–2.1 at.% (Table 1). Secondly, ICP‐OES data indicated a slight decrease of W content in the films with an increase of the deposition temperature. The oxygen content in the films is kept low, and varies between 4 at.% and 13 at.% (according to EDX) depending on the applied current density and temperature. Overall, the characterization of the morphology and composition of the films shows that different morphologies for Ni−W are accessible by simply varying the bath temperature while keeping the tungsten content almost constant. Interestingly, the W content remains between 8–12 at.% even when the concentration of tungstate is increased to 0.11 M, or the concentration of all chemicals is reduced by a factor 4 (see Supplementary Information, Tables S2 and S3).

**Table 1:** Tungsten content and current efficiency (C.E.) for Ni−W films electrodeposited from the 0.05 M Na_2_WO_4_‐containing electrolyte at −4.8 mA/cm^2^ and different bath temperatures.

T (°C)	25	35	50	65
at.% W by EDX	12.0±1.2	12.5±0.5	13.0±0.4	11.7±0.2
at.% W by ICP‐OES	12.3	11.7	10.9	9.8
C.E. (%)	11	25	47	63

**Figure 3:**
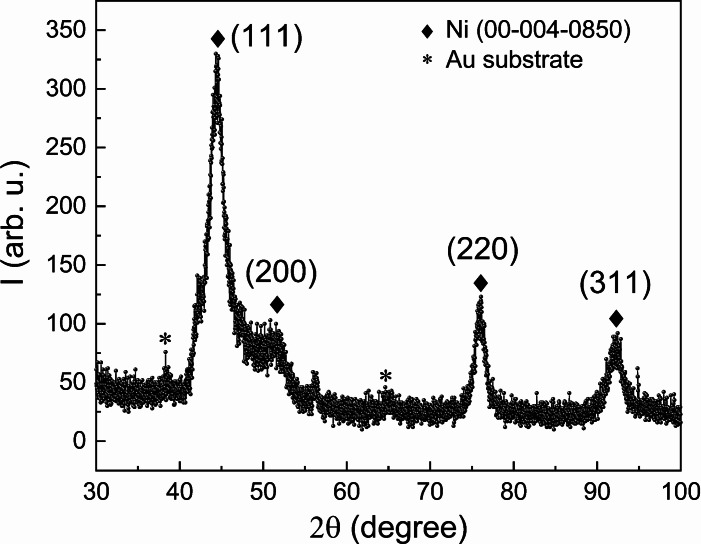
GIXRD pattern of the Ni−W film deposited at −4.8 mA/cm^2^ at 50 °C. The peaks marked with an asterisk correspond to the Au layer of the substrate.

The crystallographic structure of the Ni−W coatings was studied by grazing incidence X‐ray diffraction (GIXRD). Exemplarily, Figure 3 shows the GIXRD pattern of a Ni−W film electrodeposited at −4.8 mA/cm^2^ and 50 °C from the 0.05 M Na_2_WO_4_‐containing electrolyte. The pattern shows peaks compatible with the fcc Ni phase, which are shifted towards lower 2θ angles due to incorporation of W in the fcc Ni lattice (i. e., in the form of a solid solution). The crystal size estimated from the full width at half maximum of the (111) peak using the Scherrer equation is ~4–5 nm, indicating the nanocrystalline character of the film.

Cross‐section analyses of the films indicate that their thickness increased from ~100 nm to ~400 nm with deposition temperature at identical deposition current density and time, in agreement with the current efficiencies (C.E.) reported in Table 1. The C.E. of 63 % obtained at 65 °C is close to the value reported for the same electrolyte operated at 80 °C (61 %) for Ni−W films with 13 at.% W.^[38]^

### Electrocatalytic Activity of Ni‐W Films

The HER performance of Ni−W films with similar W content but varying morphologies was first studied by recording linear sweep voltammetry (LSV) curves in 0.5 M H_2_SO_4_ at 25 °C. Up to 200 LSV curves were recorded and a comparison between the 1^st^ and 200^th^ LSVs was made to gain insight on the durability of the films.

**Figure 4:**
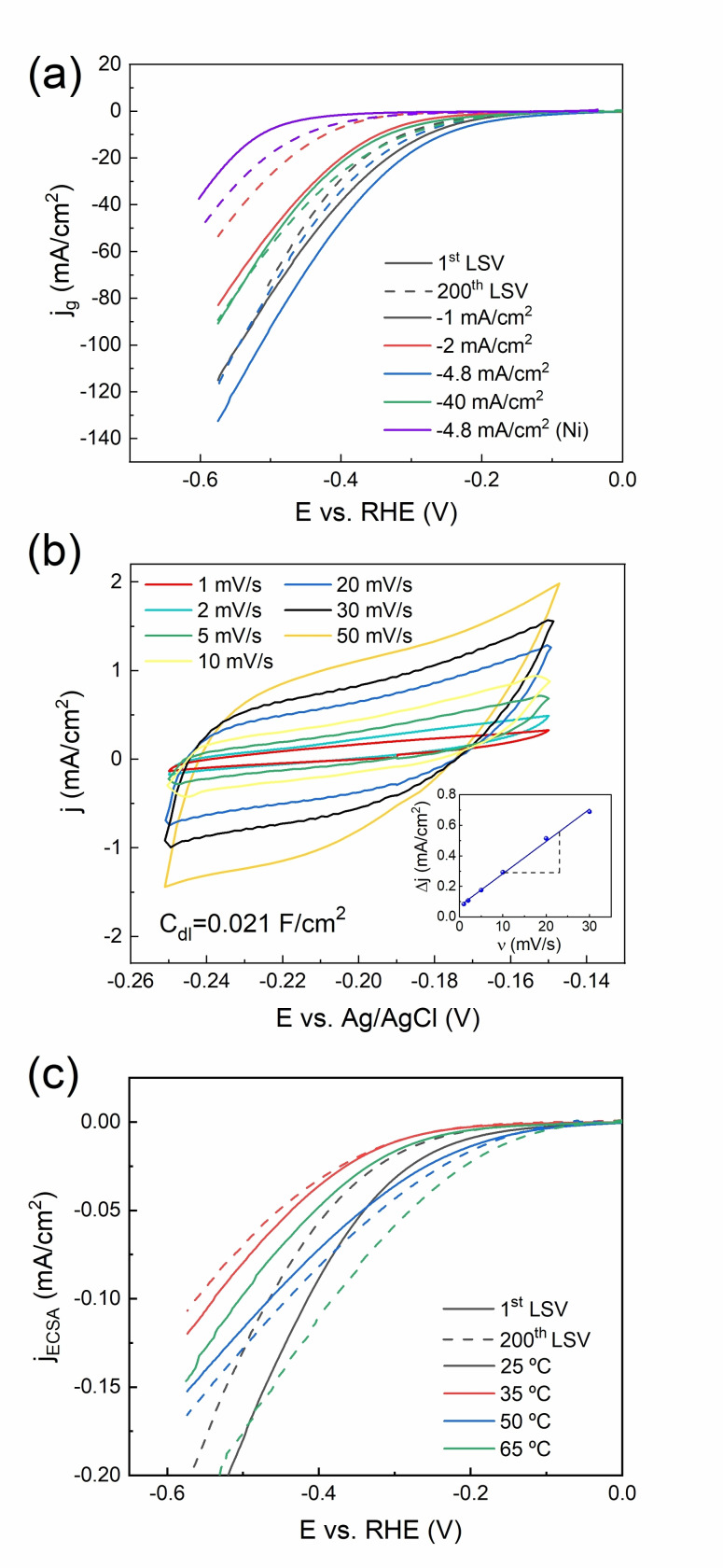
Geometric current density (j_g_) for the 1^st^ and 200^th^ LSVs of Ni−W films electrodeposited at 35 °C and different current densities (a), CVs in the capacitive regime recorded for a Ni−W film electrodeposited at −4.8 mA/cm^2^, 50 °C, and varying scan rates (b), ECSA normalized current density (j_ECSA_) for the 1^st^ and 200^th^ LSVs of Ni−W films electrodeposited at −4.8 mA/cm^2^ and different bath temperatures (c). The curve labeled as ‘−4.8 mA/cm^2^ (Ni)’ in (a) corresponds to a pure Ni film electrodeposited from the Ni−W electrolyte without tungsten salt.

**Figure 5:**
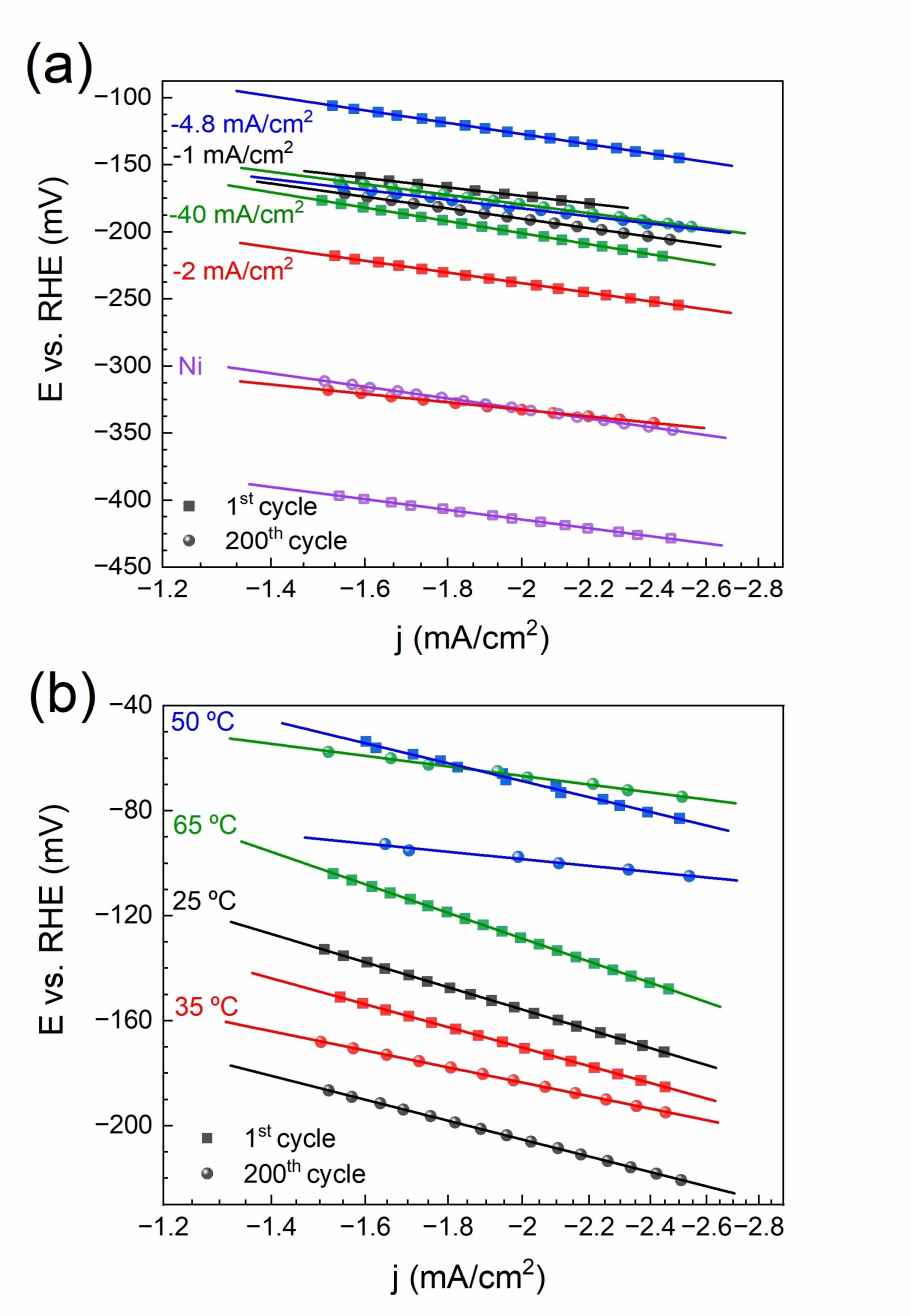
Tafel slopes determined from 1^st^ and 200^th^ LSVs for Ni−W films electrodeposited at 35 °C and different current densities (a) and −4.8 mA/cm^2^ and different bath temperatures (b). The current density is displayed in log scale.

Figure 4a shows the LSV curves recorded for Ni−W films electrodeposited from the 0.05 M Na_2_WO_4_‐containing electrolyte at 35 °C and varying current densities (−*j*=1–40 mA/cm^2^). The current was normalized by the geometric area of the films. The catalytic activity typically declines between the 1^st^ and 200^th^ cycles except for the sample electrodeposited at −40 mA/cm^2^. The Ni−W films electrodeposited at −1 mA/cm^2^ and −4.8 mA/cm^2^ show higher activity than the others. They both feature a ridge‐like morphology. Remarkably, all the tested Ni−W films outperform a pure Ni film electrodeposited from the same electrolyte without tungsten salt (purple curve). The corresponding Tafel slopes are plotted in Figure 5a. Alloying Ni with W has shown to enhance the intrinsic electrocatalytic activity over pure Ni due to alterations of the electron density in *d*‐orbitals.[^39^, ^40^]

In order to convert *j_g_* into electrochemical surface area (ECSA)‐normalized current densities (*j_ECSA_*), the ECSA of the Ni−W films was determined by CV in the range of ±50 mV around the open circuit potential (OCP). As an example, Figure 4b shows the CVs recorded on the Ni−W film electrodeposited at −4.8 mA/cm^2^ and 50 °C from the 0.05 M Na_2_WO_4_‐containing electrolyte. A linear regression of charging current vs scan rate was used to determine the double‐layer capacitance (C_dl_, see inset).^[41]^ The roughness factor (RF) values correlate well with the surface features observed by SEM and the roughness values determined by confocal microscopy (Table 2). Namely, the highest RF is found for the acicular‐grained film deposited at −4.8 mA/cm^2^ and 50 °C, giving a value 1057 times the geometrical area (Table 2).

**Table 2:** Roughness factor (RF), R_q_ and contact angle values of Ni−W coatings electrodeposited at −4.8 mA/cm^2^ from the 0.05 M Na_2_WO_4_‐containing electrolyte at different bath temperatures.

Deposition T/°C	RF	R_q_ of as‐prepared films/nm	R_q_ after 200 LSV/nm	Contact angle of as‐deposited films/°	Contact angle after 200 LSV/°
25	380	‐	‐	81	95
35	874	0.8	6.4	74	86
50	1057	1.0	2.1	85	90
65	1000	0.6	3.5	96	119

The performance of Ni−W films electrodeposited at −4.8 mA/cm^2^ for different bath temperatures was also tested (Figure 4c). The best‐performing Ni−W film is the one electrodeposited at −4.8 mA/cm^2^ and 50 °C, with an onset potential of −29 mV vs. reversible hydrogen electrode (RHE), a Tafel slope of 149 mV/dec (see Figure 5b), and an overpotential at *j_g_* =−10 mA/cm^2^ (*η_10_*) of 184 mV, as measured in the 1^st^ cycle (see Table 3). Furthermore, the 1^st^ and the 200^th^ LSV cycles are closer (i. e., showing more stable activity) compared to those derived from samples electrodeposited at 25 °C and 65 °C, hinting to fewer changes in the morphology and/or composition of the film. Overall, these results indicate that the Ni−W film grown at −4.8 mA/cm^2^ and 50 °C, with an acicular morphology, has higher activity towards HER in acidic media. Since the chemical composition of all the produced coatings is similar, as confirmed by both EDX and ICP, it can be safely deduced that the increased efficiency is primarily due to the larger surface area provided by the acicular morphology. The Tafel slopes obtained for all samples in the first cycle are around 170 mV/dec and higher, which suggests that the limiting reaction is the Volmer reaction.^[42]^

**Table 3:** Onset potential (*η*), Tafel slope (*b*), and overpotential at the geometric current density *j_g_* =−10 mA/cm^2^ (*η_10_*) of Ni−W films electrodeposited at −4.8 mA/cm^2^ and varying bath temperatures. The given values correspond to the 1^st^ and 200^th^ cycles, separated by a slash.

Bath temperature (°C)	*η* (mV)	*b* (mV/dec)	*η*_10_ (mV)
25 °C	194/106	186/156	363/241
35 °C	119/149	170/126	287/287
50 °C	29/85	149/61	184/173
65 °C	72/45	212/79	260/141

**Figure 6:**
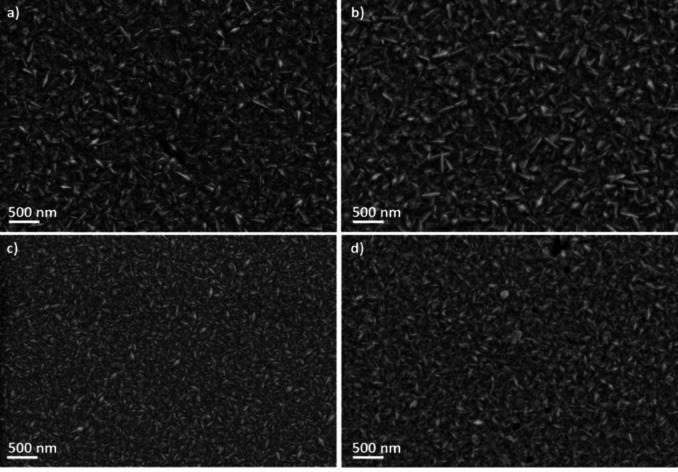
SEM images of Ni−W films deposited at −1 mA/cm^2^ and 50 °C in the as‐grown state (a) and after 200 LSVs (b), and at −4.8 mA/cm^2^ and 50 °C in the as‐grown state (c) and after 200 LSVs (d).

SEM imaging of the films after 200 LSVs in 0.5 M H_2_SO_4_ was carried out to identify the occurrence and nature of the changes. Figure 6 shows that the morphology of the samples is nearly invariant after the tests. The roughness of the films was measured before and after acquiring 200 LSVs in 0.5 M H_2_SO_4_ for correlation with the SEM observations (Table 2). For coatings grown at −4.8 mA/cm^2^, the film obtained at a bath temperature of 50 °C exhibits the highest roughness. Interestingly, this film also shows the highest RF. After 200 LSVs, the surface roughness of all films is increased but remains in the nanoscale regime. The as‐deposited films are hydrophilic, as indicated by the water contact angle values, which vary between 74° and 96° (Table 2). Following HER tests, an increase of the contact angle is observed, indicating higher hydrophobicity.

**Figure 7:**
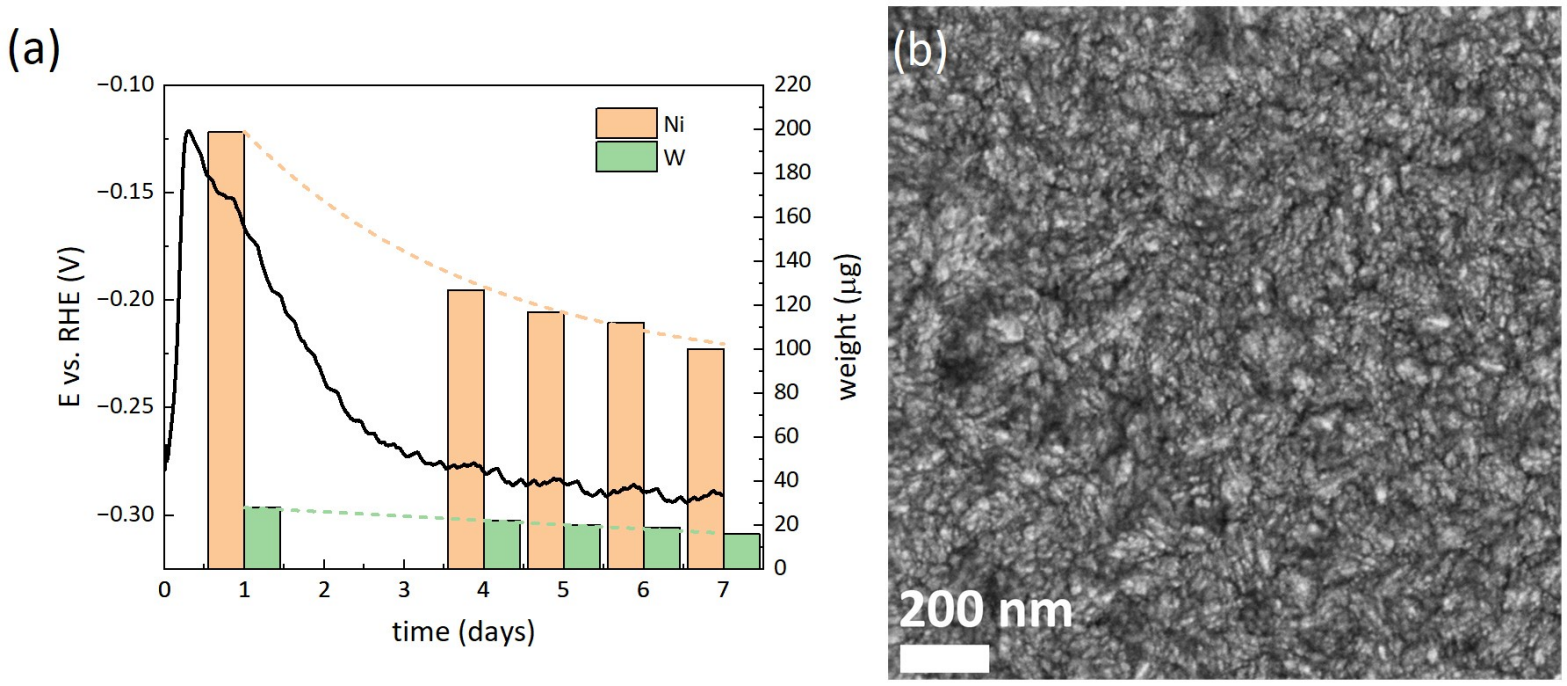
Long‐term chronopotentiometry curve recorded in 0.5 M H_2_SO_4_ at j_g_=−10 mA/cm^2^ on a Ni−W film electrodeposited at −4.8 mA/cm^2^ at 50 °C from the 0.05 M Na_2_WO_4_‐containing electrolyte (a). SEM image of the film following the 7‐day test (b). The right axis in (a) refers to the total mass of Ni and W leached from the sample in 0.5 M H_2_SO_4_, determined by ICP‐MS and displayed in a bar diagram, in addition to the dashed trendlines as a guide to the eye.

The long‐term stability of the Ni−W films was also investigated. Exemplarily, Figure 7a shows the chronopotentiometry curve of the film electrodeposited at −4.8 mA/cm^2^ and 50 °C, which was polarized for 7 days at a *j_g_* of −10 mA/cm^2^ in 0.5 M H_2_SO_4_. The potential fluctuated during the first three days but afterwards remained rather stable during the rest of the experiment, at around −0.28 V vs. RHE. The amount of Ni and W leached during the test is also included in the same plot. The results indicate preferential leaching of Ni over W, as expected. The decrease in ion concentration over time indicates material redeposition, which is rather pronounced for nickel. The sample surface shows a bluish appearance typical of tungsten oxides after the test. Nevertheless, the distinct features of the Ni−W grains are preserved, as indicated by the SEM image shown in Figure 7b. These results are in agreement with the anticipated superior corrosion resistance of Ni−W over Ni in acidic media, which has been attributed to the formation of H_0.33_O_3_W and H_2_O_4_W phases on the surface.^[43]^ In this sense, W dissolution might be hindered by the formed oxide/hydroxide species.

### Machine Learning Results

To further optimize the production of highly electrocatalytic Ni−W films, machine learning (ML) experiments were performed.

**Figure 8:**
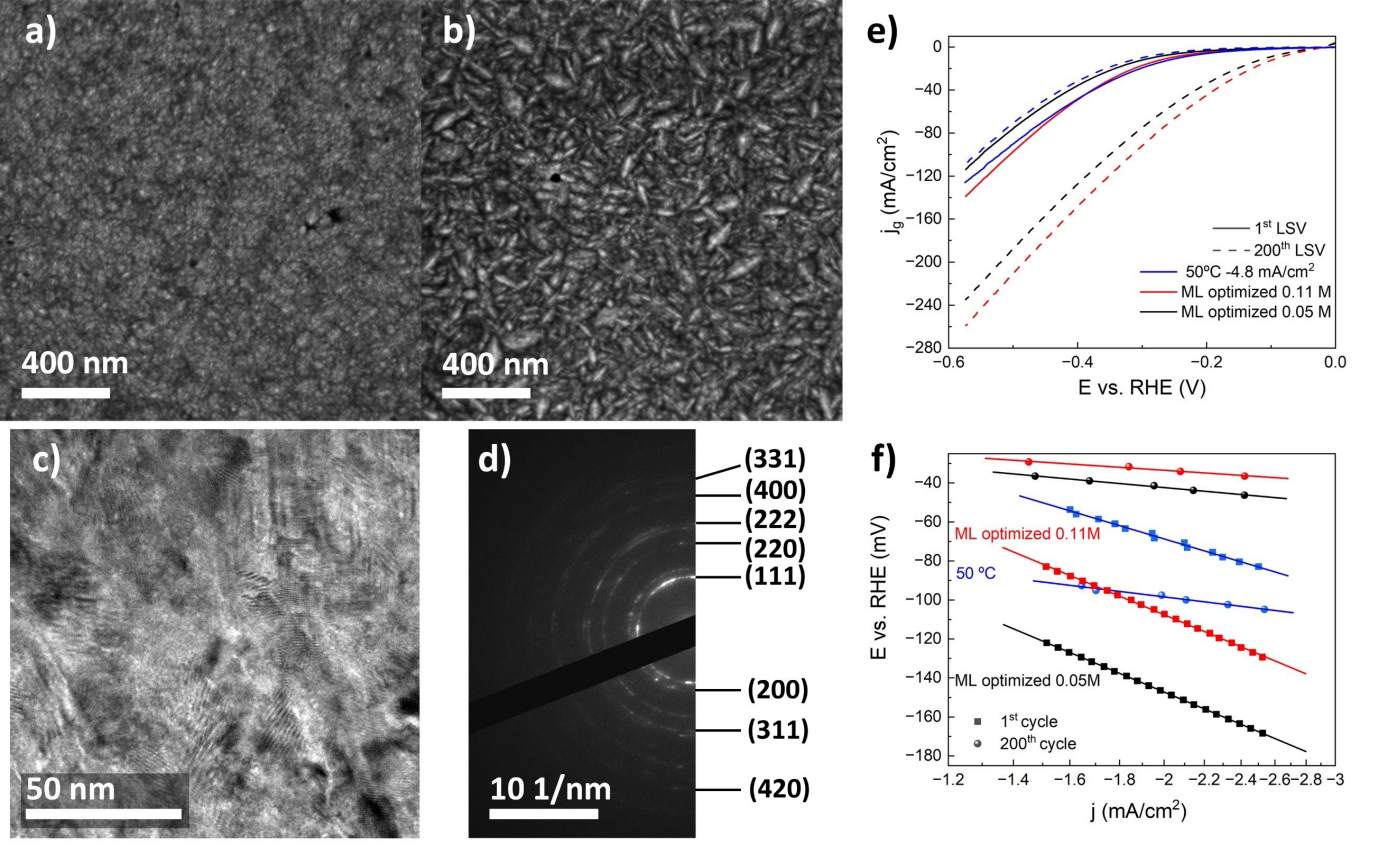
SEM images of Ni−W films deposited from the 0.05 M Na_2_WO_4_‐containing electrolyte at −9 mA/cm^2^ and 62 °C (a) and from the 0.11 M Na_2_WO_4_‐containing electrolyte at −7 mA/cm^2^ and 62 °C (b). High resolution TEM micrograph (c) and SAED pattern (d) of the film shown in (a). Corresponding 1^st^ and 200^th^ LSV curves (e) and Tafel slopes (f). The performance of the film electrodeposited at 50 °C and −4.8 mA/cm^2^ from 0.05 M Na_2_WO_4_‐containing bath is included for the sake of comparison.

The initial set of experiments that were performed before training a ML model included 24 Ni−W films with different deposition parameters and measured outputs (the whole dataset is provided in the Supplementary Information, Table S4). An initial model was trained on these 24 data points and 16 suggestions were made for the next round of active learning (see the Experimental section). The ML suggestions for plating parameters were implemented experimentally, and the catalytic performance of obtained deposits was characterized by LSV. From these data, the main catalyst characteristics were extracted and returned to the model as outputs. Since these new experiments did not lead to satisfactory results, a new ML model was generated using the initial 24 and the additional 16 data points. Using the same principles, additional deposition parameters were suggested and realized experimentally. Finally, after only one round of active learning, a set of suggested parameters (a tungstate concentration of 0.05 M or 0.11 M, a current density of *−j*=7–9 mA/cm^2^ and a bath temperature of 62 °C) led to excellent catalytic performances. The deposition time was tuned to 10 minutes ensuring that the samples could settle without cracking and peeling off. These very promising samples were then deposited again to confirm their properties (Figure 8a and b). High‐resolution transmission electron microscopy (TEM) reveals a nanocrystalline structure with an average crystal size of about 5 nm, identical to the crystal size of the Ni−W films synthesized before the ML optimization (Figure 8c). The selected area electron diffraction (SAED) pattern shows continuous diffraction rings, indicating polycrystallinity with random crystal orientation (Figure 8d). All observed diffraction rings correspond to the Ni fcc structure. The cell parameter determined from the SAED pattern is 3.51 Å, which is close to the literature value for pure Ni (3.52 Å).^[44]^ As shown in Figure 8e and f, the ML optimized samples outperform all previous samples. The measured Tafel slope for the Ni−W film deposited at −7 mA/cm^2^ and 62 °C from the 0.11 M Na_2_WO_4_‐containing electrolyte is 208 mV/dec (33 mV/dec after 200 cycles) and *η_10_* is 256 mV (88 mV after 200 cycles). A film deposited at −9 mA/cm^2^ and 62 °C from the 0.05 M Na_2_WO_4_‐containing electrolyte gave very similar results, with a Tafel slope of 209 mV/dec (45 mV/dec after 200 cycles) and *η_10_* of 284 mV (106 mV after 200 cycles).

**Figure 9:**
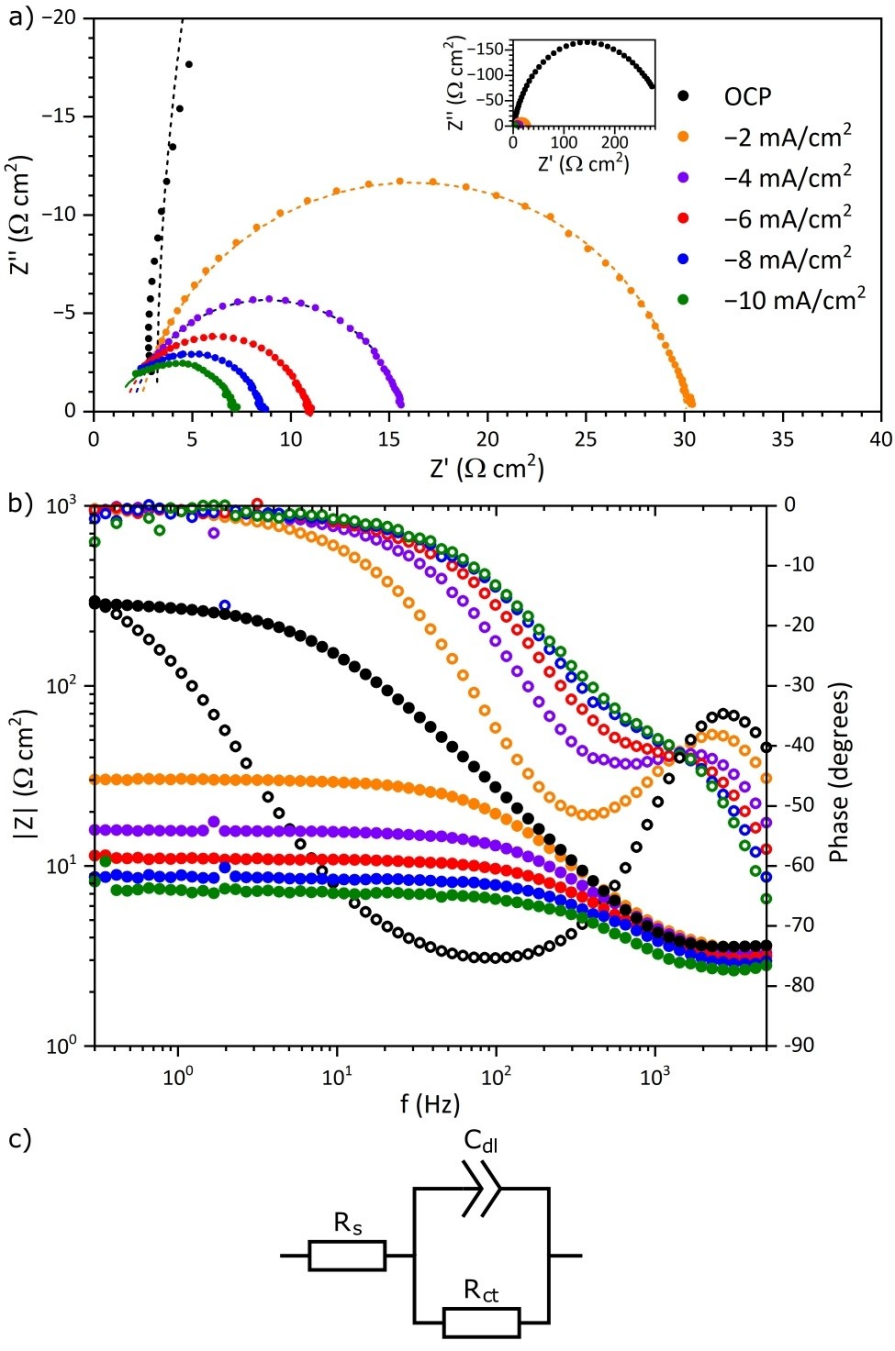
Nyquist (a) and Bode plots (b) for Ni−W film deposited from the 0.05 M Na_2_WO_4_‐containing electrolyte at −9 mA/cm2 and 62 °C, and the equivalent circuit used for the fitting (c). The markers correspond to the measured values, while the results of the fitting are displayed as dashed lines. The inset in (a) shows the full Nyquist plot for the measurement at zero current (OCP). Note that in (b), filled markers correspond to the impedance on the left axis, while empty markers correspond to the phase shift on the right axis, following the color code given by the legend in (a).

For the film electrodeposited at −9 mA/cm^2^ and 62 °C from the 0.05 M Na_2_WO_4_‐containing electrolyte, the Nyquist and Bode plots obtained from electrochemical impedance spectroscopy (EIS) during HER at different cathodic current densities reveal a capacitive behavior where the impedance decreases with the applied current density (Figure 9). A measurement at OCP was also included, yielding considerably higher impedance and reaching a phase shift close to −80° indicating strong capacitive behavior (Figure 9b).

After the fitting of the EIS data using a simplified Randles circuit as the equivalent circuit,^[45]^ and the determination of the solution resistance (R_s_), charge‐transfer resistance (R_ct_) and double‐layer capacitance (C_dl_) as described in the experimental section, a clear trend in decreasing R_ct_ with increasing absolute value of the cathodic current density is observed, showing that the surface becomes more active (Table 4). C_dl_ is rather constant around 40 μF/cm^2^, except at OCP which may indicate a slight degradation of the Ni−W film while at OCP. Very similar trends in R_ct_ with increasing overpotential were observed by Metikoš‐Huković *et al*. on sputter deposited Ni−W films in alkaline media, although obtaining considerably lower values for C_dl_ which indicate lower ECSA.^[46]^

**Table 4:** Solution resistance (R_s_), charge‐transfer resistance (R_ct_) and double‐layer capacitance (C_dl_) obtained from fitting of EIS data for the given geometric current densities (*j*).

*j* (mA/cm^2^)	R_s_ (Ω cm^2^)	R_ct_ (Ω cm^2^)	C_dl_ (μF/cm^2^)
0 (OCP)	3.2	310	86.4
−2	2.3	27.8	43.8
−4	1.9	13.8	44.0
−6	1.5	9.4	41.4
−8	1.1	7.5	36.8
−10	0.9	6.4	39.5

**Figure 10:**
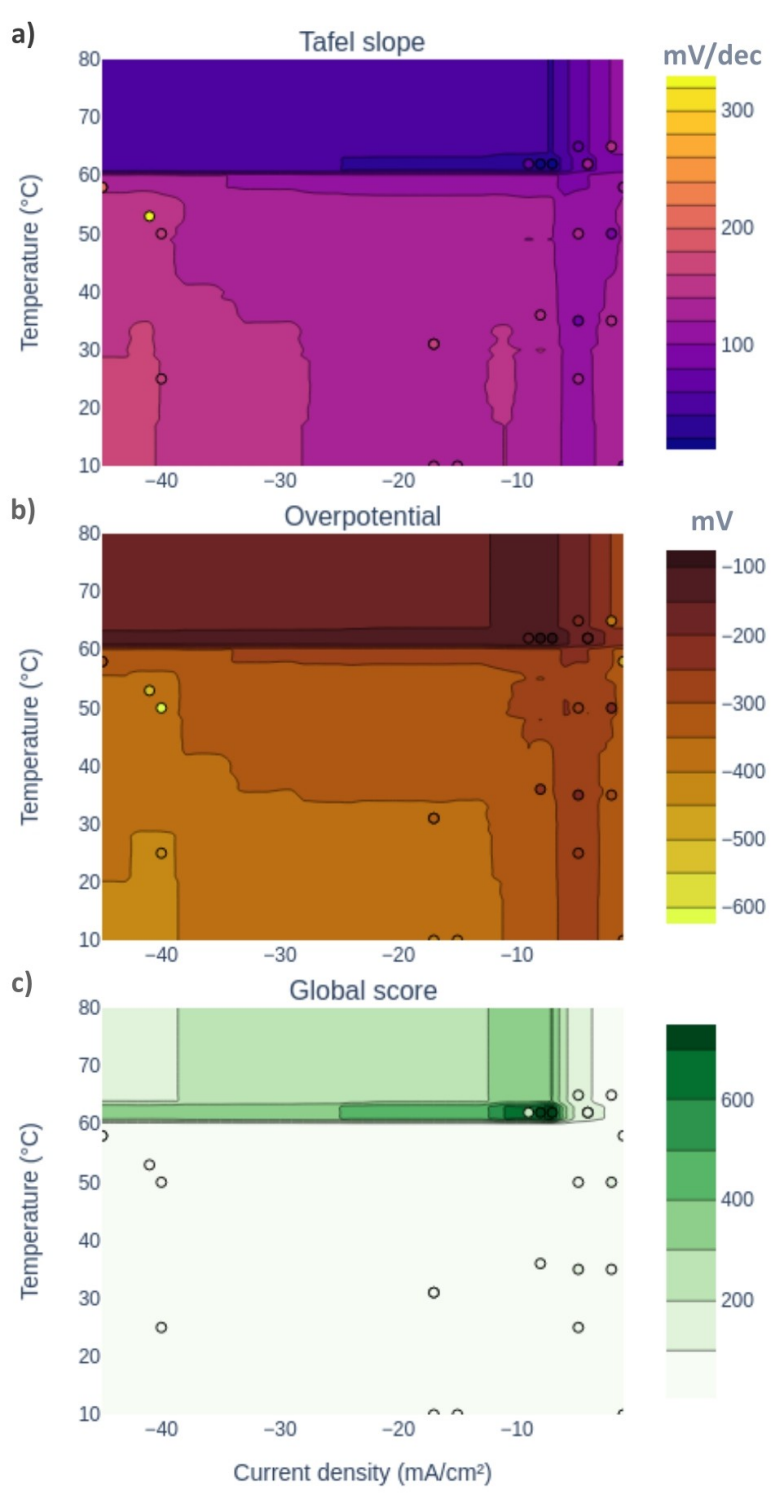
Color maps of the Tafel slope (a), overpotential (b), and global score (c) as predicted by the ML model trained on the final dataset. The 200^th^ LSV cycle is considered for the Tafel slope and overpotential. The dots represent the values for existing samples.

The final ML model allows predictions with mean absolute errors of 24 mV/dec on the Tafel slopes and 49 mV on the overpotentials, as evaluated using cross‐validation. It is also clear from the impurity‐based feature importance of the ML model that the applied current density for film deposition is the most determining factor for the model, closely followed by the bath temperature (see Figure S2 in the Supplementary Information). The deposition time and tungstate concentration of the bath are less important for the model, in accordance with the fact that these parameters are less important for the chemistry at play. The ML model also allows for visualizing the predictions on color maps, as represented in Figure 10. These can provide intuition and rules of thumbs, and a quick visual of the deposition parameters to aim for optimal performances. In this case, it is clear that temperatures slightly above 60 °C together with a current density around −10 mA/cm^2^ should be favored (Figure 10c).

### Post‐HER Characterization of the Optimized Ni‐W Films

**Figure 11:**
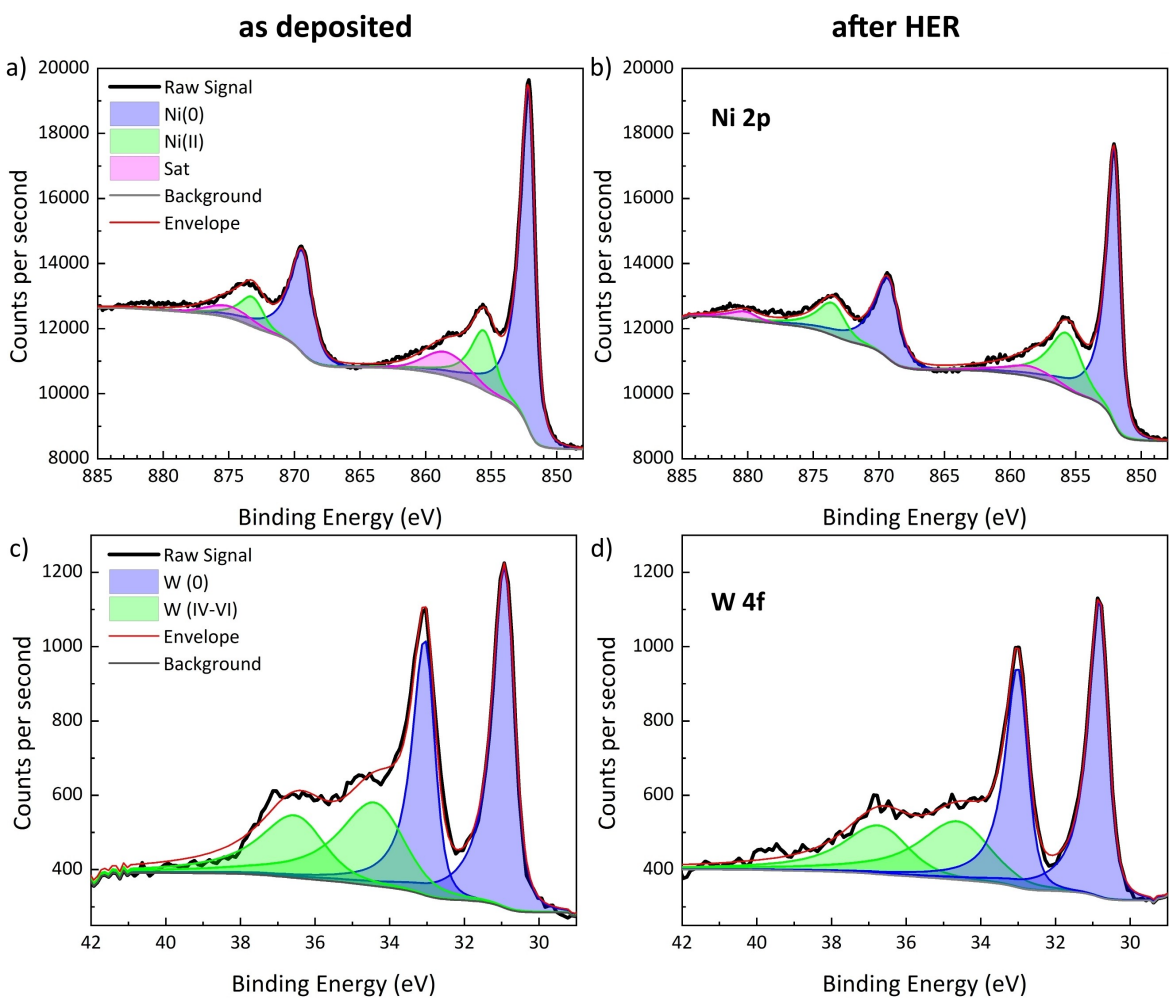
XPS core‐level spectra for Ni 2p and W 4 f for Ni−W film deposited from the 0.05 M Na_2_WO_4_‐containing electrolyte at −9 mA/cm^2^ and 62 °C as deposited (a, c) and after 200 LSV cycles of HER in 0.5 M H_2_SO_4_ (b, d).

A surface analysis by X‐ray photoelectron spectroscopy (XPS) and GIXRD of the ML optimized Ni−W films was carried out in order to elucidate on the origins of their superior performance. Initially, the XPS core‐level spectra show a predominantly metallic character, the majority of Ni 2p_3/2_ and W 4f_7/2_ emissions corresponding to zero oxidation state at 852 eV and 31 eV, respectively (Figure 11). The Ni 2p_3/2_ emissions also show peaks at 856 eV, corresponding to Ni(II), which may be assigned to surface oxidation to Ni(OH)_2_,^[47]^ in addition to a satellite peak at 858 eV. The W 4f_7/2_ emissions show a secondary doublet at 34–35 eV and 36–37 eV, compatible with an oxidation state between IV and VI_._[^43^, ^47^] After the 200 LSV cycles of HER, the ratio of Ni and W species is not changed considerably, and the W content determined by XPS remains at approx. 12 %. Regarding the oxidation states of Ni and W, only minor changes are observed. In the case of Ni, the fraction of Ni(II) increased from 22 % to 32 % during HER. This corresponds to an increase in the fraction of Ni(OH)_2_ on the surface of the Ni−W films, which is formed in the acidic medium as discussed by Arnaudova et al.,^[43]^ and accounts for the increase in overpotential observed in the long‐term durability tests in Figure 7. For W, the oxide fraction remains unchanged at 41 %, however, the average oxidation state increased slightly, as indicated by an increase in binding energy by 0.2 eV.

**Figure 12:**
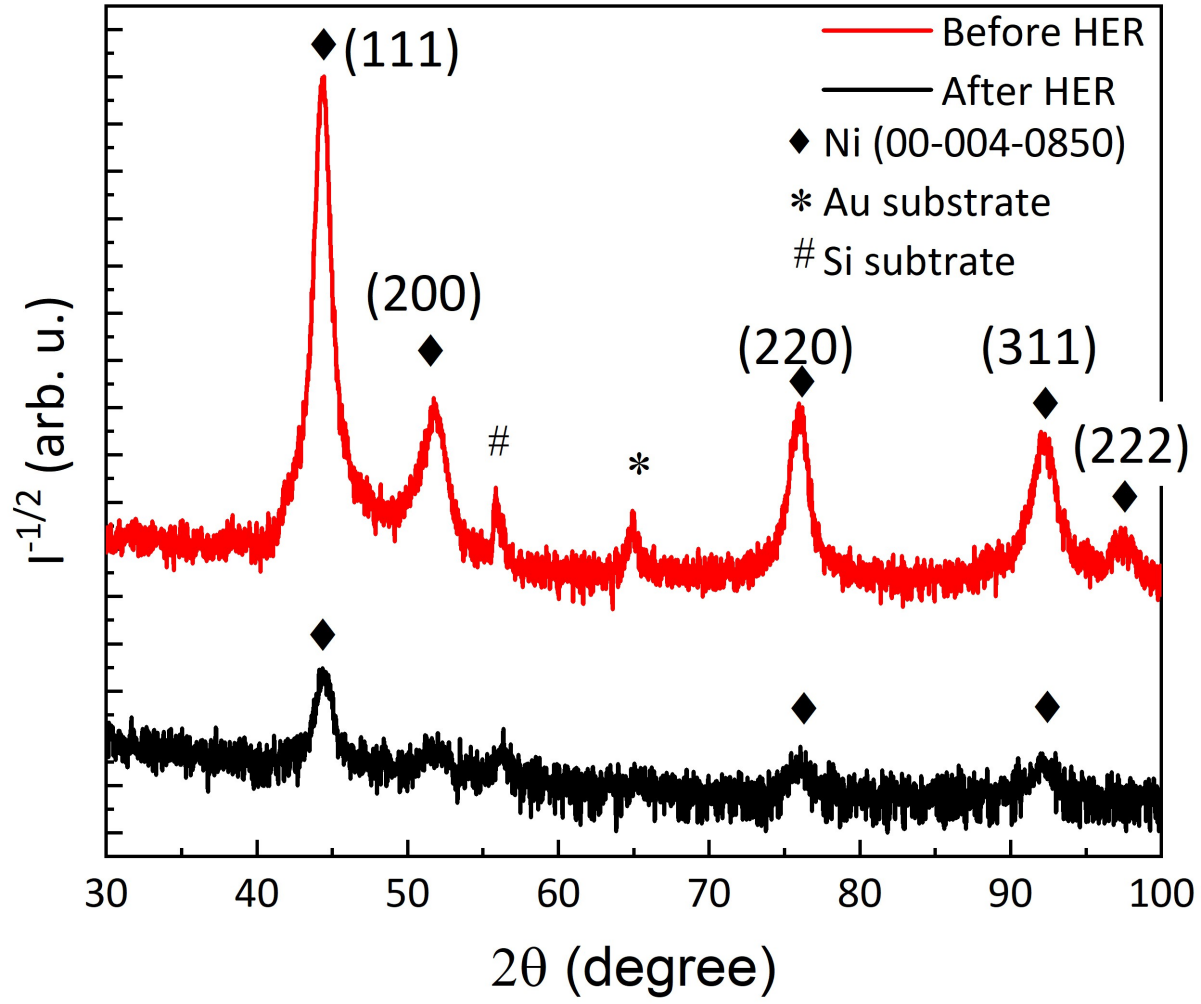
GIXRD diffractograms for Ni−W film deposited from the 0.05 M Na_2_WO_4_‐containing electrolyte at −9 mA/cm^2^ and 62 °C as deposited (red curve) and after 200 LSV cycles of HER in 0.5 M H_2_SO_4_ (black curve).

The GIXRD of the optimized Ni−W film (Figure 12) shows a very similar structure compared to one of the initial Ni−W films deposited at −4.8 mA/cm^2^ at 50 °C (cf. Figure 3). After the 200 LSV cycles of HER, the film is less crystalline, as indicated by diffraction peaks of lower intensity, while the structure is identical and shows comparable relative intensities between the peaks of the fcc phase. A larger background signal, especially at lower diffraction angles, hints at an amorphous phase, which may be the result of a metal redeposition process, as discussed earlier. Amorphous phases are reported for electrodeposited Ni‐W[^46^, ^48^] and Ni‐Mo[^49^, ^50^, ^51^, ^52^] under certain synthesis conditions, especially at low temperatures,^[50]^ and whenever oxides are at least partly involved.^[52]^ Due to their similar chemical properties and similar precursors (tungstate and molybdate), W and Mo have similar effects in electrodeposition and electrocatalysis in Ni alloys. Amorphous and nanocrystalline structures often prove to be more active at electrocatalytic reactions due to a higher density of active sites, in contrast to crystalline materials[^52^, ^53^]. This is suggested to be the main effect responsible for the considerable improvement in overpotential and Tafel slope after the 200 LSV cycles.

The HER performance of the optimal Ni−W catalysts developed throughout this study was compared with that of other Ni−W electrocatalysts reported in the literature, also obtained by electrodeposition means (Table 5). Although more nanostructured catalysts (e. g., in the form of nanoparticles) outperform the here‐produced Ni−W films, the latter show very competitive properties after 200 LSVs. Moreover, they withstand continued electrolysis at −10 mA/cm^2^ for 7 days, which is a very compelling attribute.

**Table 5:** Tafel slope (*b*) and overpotential at *j_g_* =−10 mA/cm^2^ (*η_10_*) of other Ni−W electrocatalysts obtained by electrodeposition, as reported in the literature for HER tests in 0.5 M H_2_SO_4_.

Sample	at.% W	*b* (mV/dec)	*η*_10_ (mV)	Ref.
Film	9.3	127	‐	^[54]^
Crystalline film	23	43–116^[a]^	85^[b]^	^[40]^
Nanocrystalline nanoparticulate‐like film	‐	122	205	^[7]^
Amorphous‐like/nanocrystalline coating on Cu nanowires	2	115	102	^[28]^
Nanocrystalline film	12	208/33	256/88^[c]^	This work

[a] Slopes determined in two different regions. [b] Measured at −1 mA/cm^2^. [c] Determined at 1^st^/200^th^ LSV cycle.

## Conclusions

Electroactive Ni−W films have been electrodeposited from a gluconate bath at slightly acidic pH (5.0). While keeping the tungsten content fairly constant (at around 12 at.%), surface morphology and hence, their roughness and ECSA, could be tuned by changing the applied current density and temperature. The resulting films displayed tunable HER performance in sulfuric acid in spite of having virtually the same chemical composition. Best performance was achieved for films deposited at −4.8 mA/cm^2^ and 50 °C from a 0.5 M Na_2_WO_4_‐containing electrolyte. Interestingly, the film withstands the aggressive 0.5 M H_2_SO_4_ medium under continued electrolysis at a current density of −10 mA/cm^2^ for 7 days. The implementation of a machine learning model allowed the prediction of even more electroactive Ni−W films, which should be deposited at a slightly more negative current densities (−*j*=7–9 mA/cm^2^) and at a temperature of 62 °C. Their higher electrocatalytic activity was experimentally verified, while saving material resources and laboratory time. The high stability and the considerable improvement during 200 LSV cycles of HER was related to a restructuring of the surface by redeposition of Ni and W, resulting in an amorphous‐like metallic surface with an increased active site density.

## Experimental Section

*Electrochemical synthesis of Ni‐W films*: The Ni−W films were electrodeposited in a double‐jacketed three‐electrode cell connected to a 302 N Autolab potentiostat/galvanostat. The reference electrode (RE) used for the measurements was a double junction Ag/AgCl reference electrode (Metrohm AG) with 3 M KCl inner solution and 1 M Na_2_SO_4_ outer solution. The counter electrode (CE) was a spiral of platinum wire. Ni−W films were grown on metallized silicon (111) substrates with e‐beam evaporated Ti (10 nm)/Au (90 nm) adhesion/seed layers.

The electrolyte was reproduced from Bera *et al*.^[38]^ and contained 0.11 M NiSO_4_⋅7 H_2_O, 0.05 M Na_2_WO_4_⋅2 H_2_O, 0.5 M sodium gluconate and 0.65 M H_3_BO_3_ in Milli‐Q (MQ)‐water, unless otherwise stated. Namely, in some cases the tungstate concentration was increased to 0.11 M, or the concentration of all chemicals was 4‐fold decreased. The bath pH was adjusted to 5.0 with sulfuric acid. Depositions were run between 25 °C and 65 °C instead of 80 °C, as used in Bera′s study. At 65 °C, the solution was periodically refilled with water to account for losses due to evaporation. The temperature of the bath was controlled by circulating water through the external vessel′s jacket using a thermostat from Julabo. Before deposition, the bath was de‐aerated with N_2_ gas by bubbling it through the electrolyte and a blanket of N_2_ was maintained on top of the solution during deposition. The coatings were deposited galvanostatically at current densities ranging from −1 to −40 mA/cm^2^ and varying temperatures, under agitation using a magnetic bar (ω=100 rpm). Following deposition, the samples were MQ‐water rinsed and dried in air. Prior to the deposition of the films, the electrolyte was characterized by CV. The potential was scanned from an initial value at which current was not recorded towards cathodic values, and then it was reversed towards anodic potentials. The scan rate was 50 mV/s.

*Electrochemical activity*: The electrochemical activity of the as‐deposited Ni−W coatings towards HER was studied in the same set‐up used for the growth of the films. The samples were tested in 0.5 M H_2_SO_4_ at 25 °C, using a graphite bar as a CE and the previously electrodeposited Ni−W films as the working electrode (WE). The potentials were converted to the RHE according to (Equation 1:

(1) E(RHE)=E(Ag/AgCl)+0.210V+0.059VpH

200 cycles of LSV curves were run from OCP towards −0.6 V vs RHE to investigate catalyst activity. The Tafel slope (*b*), the onset overpotential (*η*), and the overpotential at −10 mA/cm^2^ (*η_10_*) were determined. To determine the ECSA of the films, the capacitance method was used, which consists of cycling the electrocatalysts in the non‐faradaic region at varying scan speeds.^[41]^ For this purpose, CVs were recorded at OCP ±50 mV from negative to positive potentials at scan speeds ranging from 1 to 50 mV/s. The C_dl_ was determined from the voltammetries at OCP according to (Equation (2)) while C_s_ of 20 μF/cm^2^ was obtained from literature for Ni−W.^[55]^ The RF was obtained from (Equation 3:

(2) $$C_{dl}=\Delta \left(j_{a}-j_{c}\right)/2\nu$$

(3) $$RF=\;\frac{C_{dl}}{C_{s}}$$

where Δj is the difference between anodic and cathodic current densities, and ν is the scan rate.

EIS was performed on ML optimized Ni−W films in a frequency range from 15 kHz to 300 mHz, using an amplitude of 100 μA, for the given current densities of 0 (corresponding to OCP), 2, 4, 6, 8 and 10 mA/cm^2^. Fitting of the data was performed using Zfit for Matlab^[56]^ using the equivalent circuit given in Figure 9c with a constant phase element (CPE) representing C_dl_. C_dl_ was then determined from the parameters obtained for the CPE according to Equation 4 given by Brug *et al*.^[57]^

(4) $$C_{dl}={\left(\frac{CPE}{{\left(\frac{1}{R_{s}}+\frac{1}{R_{ct}}\right)}^{1-n}}\right)}^{\frac{1}{n}}$$

where n is the exponent of the CPE.

*Morphological, compositional, and structural characterization*: A Zeiss Merlin field emission SEM equipped with an EDX detector was used for the characterization of the morphology and composition of the coatings. Morphological observations were carried out at 8 keV, whereas EDX analyses were made at 15 keV. Due to overlapping of Si (K_α_=1.740 eV, K_β_=1.837 eV) and W (M_α_=1.775 eV, M_β_=1.838 eV) peaks in EDX patterns, the W content can be largely overestimated. The contribution of Si is automatically subtracted by the INCA software to provide the real W content in the films. In this quantification, oxygen was disregarded, so only Ni and W elements were considered. TEM was performed on a cross‐section of the Ni−W film whose synthesis parameters were optimized by ML, using a JEOL 2011 TEM at 200 keV in bright field and diffraction mode for collection of SAED patterns. Image analysis was performed using ImageJ. The GIXRD patterns were obtained with a Philips X′Pert diffractometer in the 2θ range of 30–110° using Cu K_α_ radiation. XPS was performed using a SPECS PHOIBOS 150 hemispherical analyzer with Al K_α_ radiation, and fitting of the data was performed using CasaXPS.^[58]^ The current efficiency of the electrodeposition process was estimated using Faraday′s law of electrolysis and the masses of Ni and W determined by ICP‐OES. For this purpose, the films were digested in a solution of concentrated HF, HCl and HNO_3_. The resulting solutions were then analyzed by ICP‐OES on an Agilent model 5900 spectrometer to determine the actual amount of W and Ni in the coatings. The roughness before and after the 200 LSVs in acidic medium was determined by the average of the root mean square deviation (R_q_) obtained from three different areas of the coating, using a Leica DCM 3D confocal microscope. The thickness was determined from cross‐section SEM imaging of some selected coatings. The wettability of the Ni−W films was tested using a Drop Shape Analyzer DSA 100 from KRÜSS, in which 4 ml water droplets were placed on the film surface at a speed of 1185 ml/min.

To investigate the long‐term stability of the films in 0.5 M H_2_SO_4_ under constant current application, a two‐compartment three‐electrode cell was employed. The Ni−W sample served as the working electrode, a reversible hydrogen electrode (HydroFlex from Gaskatel) acted as the reference electrode, and a Pt mesh was used as the counter electrode. The cathodic and anodic compartments, each with a volume of 250 ml, were separated by a proton exchange Nafion membrane. The membrane effectively prevents the contamination of the Ni−W electrode surface with anodic reaction products formed during extended tests. A constant current density of −10 mA/cm^2^ was applied to the working electrode, and the potential response was recorded continuously for up to 7 days (168 h). To assess the concentration of leached metal ions, 10 mL of the electrolyte were extracted from the cathodic compartment on the 1^st^, 4^th^, 5^th^, 6^th^, and 7^th^ days of the experiment for inductively coupled plasma mass spectrometry (ICP‐MS) in an Agilent model 7900 spectrometer.

*Machine learning*: The ML model is based on a Random Forest as implemented in the scikit‐learn package.^[59]^ The training was repeated 50 times, each time including 90 % of the data. The predicted outputs are then the average of the predictions of those 50 submodels while their standard deviation accounts for the uncertainty of the model. Cross‐validation is performed by training the ML model on all but one data points, that is used as the test set. An average of the mean absolute error over all the possible test sets (i. e., over all of the data points) indicates the performances of the ML model trained on the whole dataset for predictions on unseen deposition parameters. The inputs of the models are the tungstate concentration of the bath, the deposition current density, temperature, and time (i. e., the parameters that the experimentalists performing the deposition control). The outputs are the Tafel slope and overpotential at −10 mA/cm^2^, each determined at the 1^st^, 5^th^, 10^th^, 50^th^, 100^th^, 150^th^, and at the 200^th^ LSV cycles. While *η_10_* is easily determined, the Tafel slopes were all obtained at −1 mA/cm^2^ using an in‐house Python script automating the task for all the LSV curves of each sample (cf. Figure S4).

While the ML model can be used to predict the Tafel slopes and overpotentials (outputs) for any given deposition parameters (inputs), the optimization of multiple outputs through active learning is more easily done by first defining a global score for each sample. Indeed, this avoids having to optimize all the outputs at once, particularly in the case where this task cannot be achieved (e. g., optimizing one output degrades the performance on the others). To this purpose, we define the evolution of the Tafel slope and overpotential between the 1^st^ and 200^th^ cycles as:

(5) $$Evolution=\frac{\left|X^{1}\right|-\left|X^{200}\right|}{\left|X^{1}+X^{200}\right|}$$

where *X* ^*1*^ and *X* ^*200*^ may be either *b* or *η_10_* at the 1^st^ and the 200^th^ cycle, respectively. Since the absolute values of the Tafel slope and overpotential should be as small as possible, this definition of evolution is positive if catalytic activity improves, and negative if activity decreases. The global score, that can be used to rank the deposited samples, is arbitrarily defined as:

(6) $$Score={\left(\frac{1+Evolution\left(b\right)}{b^{200}}\right)}^{\alpha }{\left(\frac{1+Evolution\left(\eta_{10}\right)}{\eta_{10}^{200}}\right)}^{\beta }$$

where *α* and *β* can be tuned to give more importance to the Tafel slope or to the overpotential, respectively. This definition of the global score leads to large values for samples with low *b* and *η_10_* at the 200^th^ LSV cycle. Their evolutions are also included in the numerator: a positive evolution leads to a larger score. Adding 1 to each numerator allows for dealing with strictly positive scores and avoids a situation where a zero evolution, which is not necessarily bad, leads to a zero score regardless of the Tafel slope and overpotential values. In this work, we decided to give the same importance to both the Tafel slope and overpotential in the global score, i. e., set *α=β=*1.

The evolutions and global score are also used as outputs of the ML model to be trained on. However, since the model does not conserve the mathematical relationship between the predicted Tafel slopes, overpotentials, evolutions and scores, for final predictions we use the predicted Tafel slopes and overpotentials to compute the evolutions and global score. This is justified by the fact that the evolutions and score predicted by the model and those computed from Equations (5) and (6) using the predicted Tafel slopes and overpotentials are strongly correlated and a correct ranking of deposited samples can be done through either the predicted or computed scores, see Figure S3 in Supplementary Information.

For the active learning process, the inputs space (bath composition, current density, temperature, and deposition time) is sampled homogeneously with the two baths used for training (containing either 0.05 M or 0.11 M Na_2_WO_4_), 45 current densities ranging from −1 to −45 mA/cm^2^, 71 temperatures ranging from 10 to 80 °C, and 36 deposition times ranging from 5 to 40 minutes. In total, 230,040 possible sets of deposition parameters are therefore evaluated using the latest ML model. Then, candidate deposition parameters were suggested based on 1) the optimization of the Tafel slope and overpotential at the 200^th^ cycle, their evolution, and the global score, 2) the maximization of the prediction uncertainties for these quantities, and 3) the optimization of the acquisition function defined as the sum (or difference, in case minimization is needed) of the predictions and uncertainties. This allowed the procedure to make (sometimes overlapping) suggestions in batches instead of one at a time. In 1), the active learning is performed in the so‐called exploitation mode, which aims at reaching the optimal performances, while in 2) the exploration mode allows for improving the model in the most efficient way. Using the acquisition function, in 3), allows a trade‐off between the two modes of active learning.

## Conflict of Interests

The authors declare no conflict of interest.

1

## Supporting information

As a service to our authors and readers, this journal provides supporting information supplied by the authors. Such materials are peer reviewed and may be re‐organized for online delivery, but are not copy‐edited or typeset. Technical support issues arising from supporting information (other than missing files) should be addressed to the authors.

Supporting Information

## Data Availability

The data that support the findings of this study are openly available in CORA RDR at https://doi.org/10.34810/data1750.
