# Supplementary material for: Hydrogen Evolution Reaction of Electrodeposited Ni‐W Films in Acidic Medium and Performance Optimization Using Machine Learning
Source: ChemSusChem. 2024 Nov 13;18(5):e202400444. doi: 10.1002/cssc.202400444 (PMC11874652; doi:10.1002/cssc.202400444)
Supplement: Supplementary file 1 — Supporting Information [file CSSC-18-e202400444-s001.pdf]

ChemSusChem

Supporting Information

Hydrogen Evolution Reaction of Electrodeposited Ni-W Films in Acidic Medium and Performance Optimization Using Machine Learning

Roger de Paz-Castany, Konrad Eiler, Aliona Nicolenco, Maria Lekka, Eva García-Lecina, Guillaume Brunin, Gian-Marco Rignanese, David Waroquiers, Thomas Collet, Annick Hubin, and Eva Pellicer*

Supporting Information file

Hydrogen evolution reaction of electrodeposited Ni-W films in acidic medium and performance optimization using machine learning

Roger de Paz-Castany,[a] Konrad Eiler,[a] Aliona Nicolenco,[b] Maria Lekka,[b] Eva García-Lecina,[b] Guillaume Brunin,[c] Gian-Marco Rignanese,[c] David Waroquiers,[c] Thomas Collet,[d] Annick Hubin,[d] Eva Pellicer*[a]

[a] R. de Paz-Castany, K. Eiler, E. Pellicer
Physics department
Universitat Autònoma de Barcelona
Campus de la UAB, 08193 Bellaterra (Cerdanyola del Vallès), Spain
E-mail: Eva.Pellicer@uab.cat

[b] A. Nicolenco, M. Lekka, E. García-Lecina
CIDETEC, Basque Research and Technology Alliance (BRTA),
Pº Miramón 196, 20014 San Sebastián, Spain

[c] G. Brunin, G.-M. Rignanese, D. Waroquiers
Matgenix
A6K Advanced Engineering Centre Sq. des Martyrs 1, 6000 Charleroi, Belgium

[d] T. Collet, A. Hubin
SURF department
Vrije Universiteit Brussel, Pleinlaan 2, 1050 Brussels, Belgium

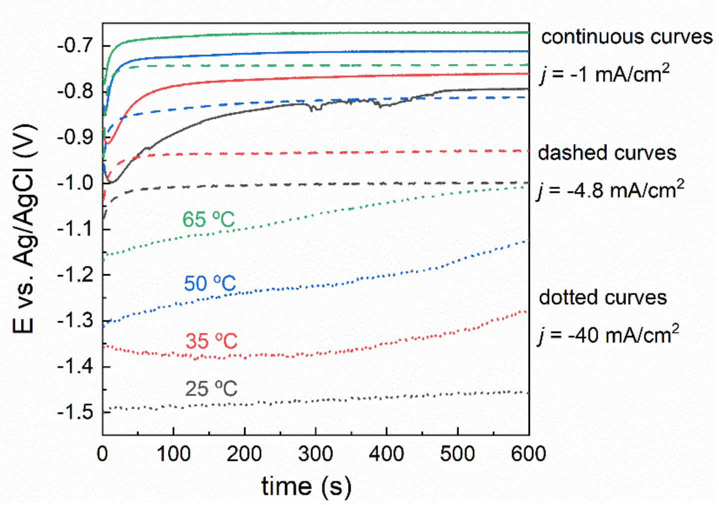

Figure S1. *E*-t curves recorded during the deposition of the Ni-W films from the electrolyte containing 0.11 M $NiSO_4 \cdot 7\ H_2O$, 0.05 M $Na_2WO_4 \cdot 2\ H_2O$, 0.5 M $NaC_6H_{11}O_7$, 0.65 M H_3BO_3 at pH = 5.0 and the indicated bath temperature and current density. The temperature color code is the same for all current densities.

Table S1. Tungsten content in Ni-W films, determined by EDX measurements, electrodeposited from 0.11 M $NiSO_4 \cdot 7\ H_2O$, 0.05 M $Na_2WO_4 \cdot 2\ H_2O$, 0.5 M $NaC_6H_{11}O_7$, 0.65 M H_3BO_3, pH = 5.0, at the indicated bath temperature and current density.

T / ºC	−1 mA/cm^2	−40 mA/cm^2
25	5.4*	10.7
35	11.6	9.3
50	11.2	13.3
65	9.4	12.9

*Not reliable due to the low film thickness.

Table S2. Tungsten content in Ni-W films, determined by EDX measurements, electrodeposited from 0.0275 M $NiSO_4 \cdot 7\ H_2O$, 0.0125 M $Na_2WO_4 \cdot 2\ H_2O$, 0.125 M $NaC_6H_{11}O_7$, 0.1625 M H_3BO_3, pH = 5.0, at the indicated bath temperature and current densities.

Deposition conditions	at.% W
35 ºC, –40 mA/cm^2	9.6
50 ºC, –1mA/cm^2	9.0
50 ºC, –4.8 mA/cm^2	8.6
65 ºC, –1 mA/cm^2	7.3
65 ºC, –40 mA/cm^2	12.8

Table S3. Tungsten content in Ni-W films electrodeposited from 0.11 M $NiSO_4 \cdot 7\ H_2O$, 0.11 M $Na_2WO_4 \cdot 2\ H_2O$, 0.5 M $NaC_6H_{11}O_7$, 0.65 M H_3BO_3, pH = 5.0, as a function of the stirring conditions, at the indicated current densities and bath temperature.

at %W	−1 mA/cm^2	−4.8 mA/cm^2	−40 mA/cm^2
25 ºC	No deposition	11.3 ± 0.8	8.1 ± 0.7
35 ºC	7.7± 0.3	10.3 ± 0.5	9 ± 1
50 ºC	10.0 ± 0.3	11.0 ± 0.4	10.9 ± 0.2
65 ºC	8.5 ± 0.6	10.5 ± 0.7	11.4 ± 0.2

Table S4. Detailed results for each sample deposited and characterized. The tungstate concentration of the bath, deposition current density, temperature, and time are first reported. Then, the Tafel slope at −1 mA/cm², as computed using an in-house Python script, for the 1st, 5th, 50th, 100th, 150th, and 200th cycles are given. The overpotential at −10 mA/cm² are also provided. The Tafel slope and overpotential evolutions between the 1st and 200th cycles as well as the global score are finally reported. The samples initially used for the first ML model are those from samples IV to A11. The samples that have been suggested after the first round of active learning are labeled from A to S. The last round of active learning provided the candidates from A3 to Q3. (See next three pages).

Sample name	Na_2WO_4 (M)	j (mA/cm^2)	T (°C)	time (min)	Tafel (mV/dec) cycle 1	Tafel (mV/dec) cycle 5	Tafel (mV/dec) cycle 50	Tafel (mV/dec) cycle 100	Tafel (mV/dec) cycle 150	Tafel (mV/dec) cycle 200	E (mV) at -10 mA/cm^2 cycle 1	E (mV) at -10 mA/cm^2 cycle 5	E (mV) at -10 mA/cm^2 cycle 50	E (mV) at -10 mA/cm^2 cycle 100	E (mV) at -10 mA/cm^2 cycle 150	E (mV) at -10 mA/cm^2 cycle 200	Tafel slope evolution	Potential evolution	Score
IV	0,05	-38	25	10	183,16	170,74	183,40	190,57	193,34	195,38	-346,56	-363,63	-415,11	-417,47	-417,36	-425,50	-0,03	-0,10	10,45
V	0,05	-30	25	10	203,24	158,69	110,90	106,84	135,19	125,14	-328,04	-312,28	-262,35	-263,06	-319,09	-348,47	0,24	-0,03	27,53
VII	0,05	-4,8	25	10	202,07	178,82	144,61	118,58	112,81	114,53	-362,61	-375,00	-273,95	-248,10	-239,37	-241,00	0,28	0,20	55,56
VIII	0,05	-1	25	30	193,47	145,81	137,92	135,77	155,17	161,09	-340,37	-288,81	-322,95	-363,32	-399,67	-409,29	0,09	-0,09	15,03
XVII	0,05	-3	35	10	185,11	157,99	82,08	84,48	129,35	127,98	-346,12	-333,14	-234,13	-233,63	-276,49	-332,12	0,18	0,02	28,39
XV	0,05	-4,8	50	10	103,16	53,70	28,92	17,39	21,56	33,87	-183,73	-187,10	-141,03	-143,68	-154,75	-173,07	0,51	0,03	264,49
XIX	0,05	-1	35	40	170,90	131,90	144,66	149,75	150,46	152,39	-341,53	-328,56	-330,24	-343,72	-353,40	-363,42	0,06	-0,03	18,50
XIV	0,05	-4,8	35	10	166,25	122,00	101,62	99,00	106,38	105,85	-286,55	-274,01	-251,83	-267,51	-286,80	-286,90	0,22	0,00	40,21
X	0,05	-2	35	10	218,42	156,56	130,65	155,98	164,26	159,86	-347,07	-311,78	-276,66	-339,65	-418,10	-422,36	0,15	-0,10	15,43
IX	0,05	-1	50	30	186,03	153,63	74,74	98,12	190,81	195,18	-304,85	-288,09	-211,21	-229,73	-284,30	-317,67	-0,02	-0,02	15,42
XX	0,05	-1	65	30	179,82	162,37	167,29	167,11	169,43	172,43	-344,54	-347,37	-347,86	-358,31	-370,16	-384,70	0,02	-0,06	14,54
XXII	0,05	-3	65	10	203,70	166,49	142,81	118,27	119,03	125,73	-330,86	-337,47	-281,45	-259,40	-253,01	-264,11	0,24	0,11	41,42
XXIII	0,05	-4	65	10	210,78	169,48	118,50	90,52	74,41	69,57	-338,72	-345,99	-254,92	-222,07	-207,71	-201,97	0,50	0,25	134,09
XXIV	0,05	-4,8	65	10	167,12	109,70	61,07	56,98	55,22	56,88	-260,19	-250,39	-151,79	-139,95	-129,80	-140,85	0,49	0,30	241,68
XXVII	0,05	-40	65	5	177,59	138,30	149,49	147,86	146,15	144,19	-287,08	-282,09	-277,83	-275,06	-273,40	-274,84	0,10	0,02	28,46
A1	0,11	-40	25	5	213,48	154,12	175,65	160,18	155,67	158,51	-335,74	-348,84	-389,26	-415,16	-420,13	-428,40	0,15	-0,12	14,85
A2	0,11	-4,8	25	10	182,99	129,63	118,78	114,45	115,25	125,73	-294,24	-298,18	-270,55	-258,74	-253,69	-259,06	0,19	0,06	38,71
A4	0,11	-2	35	30	178,00	97,46	43,97	87,79	134,82	134,02	-266,29	-251,78	-178,47	-224,64	-283,48	-298,01	0,14	-0,06	26,96
A5	0,11	-4,8	35	10	166,30	116,90	50,65	47,59	58,85	79,00	-274,17	-270,99	-188,26	-176,87	-188,90	-207,90	0,36	0,14	93,91
A7	0,11	-2	50	30	202,86	154,63	47,04	41,54	54,72	86,15	-312,46	-307,03	-180,75	-163,40	-181,86	-212,93	0,40	0,19	91,03
A8	0,11	-4,8	50	10	171,99	94,96	28,44	40,95	108,94	131,49	-283,44	-251,50	-141,62	-158,29	-231,70	-272,22	0,13	0,02	32,30
A9	0,11	-40	50	30	253,14	215,96	184,24	164,75	155,23	154,65	-684,00	-693,00	-610,91	-595,00	-596,31	-608,95	0,24	0,06	13,95

A10	0,11	-2	65	30	154,66	149,20	95,99	133,90	143,54	138,04	-281,20	-265,14	-209,99	-275,97	-379,47	-388,69	0,06	-0,16	16,54
A11	0,11	-4,8	65	10	183,92	128,43	62,35	54,51	49,08	69,86	-289,80	-285,30	-183,56	-167,82	-171,95	-213,32	0,45	0,15	112,06
A	0,05	-38	46	5	194,72	145,19	128,72	121,24	105,80	99,25	-348,48	-368,51	-315,75	-286,09	-279,26	-284,40	0,32	0,10	51,69
C	0,05	-17	46	15	179,46	139,70	115,34	104,57	101,53	104,44	-365,82	-386,90	-355,69	-342,97	-334,52	-333,07	0,26	0,05	38,05
D	0,11	-17	31	10	221,22	139,98	126,66	119,55	118,10	119,69	-351,64	-365,35	-360,88	-366,60	-370,08	-366,57	0,30	-0,02	28,97
E	0,11	-45	58	3	187,00	145,85	128,43	130,17	101,18	109,35	-339,99	-347,55	-315,28	-289,68	-280,11	-278,09	0,26	0,10	45,66
F	0,11	-17	31	10	202,78	145,96	149,93	152,43	130,46	157,72	-357,51	-367,37	-370,05	-360,31	-350,04	-341,32	0,12	0,02	21,38
G	0,05	-17	10	15	182,26	149,86	108,23	114,44	113,21	113,58	-390,34	-423,77	-406,29	-401,97	-402,77	-403,59	0,23	-0,02	26,43
I	0,05	-2	38	40	192,34	89,84	118,17	113,59	125,29	130,54	-343,28	-264,62	-331,07	-394,10	-448,31	-456,21	0,19	-0,14	17,18
J	0,11	-15	10		211,50	162,42	139,71	136,83	131,51	135,16	-359,86	-373,94	-374,67	-380,91	-385,28	-389,17	0,22	-0,04	22,29
K	0,11	-1	10	40	204,06	128,28	117,54	75,74	74,62	77,00	-358,87	-334,90	-343,92	-325,23	-326,94	-331,80	0,45	0,04	59,06
M	0,05	-17	38	15	194,53	144,73	120,52	118,77	116,62	114,57	-392,61	-427,27	-392,12	-378,90	-367,51	-362,15	0,26	0,04	31,56
N	0,05	-45	58	3	193,82	168,97	131,51	127,35	121,64	120,07	-373,81	-397,29	-409,87	-421,26	-408,83	-401,10	0,23	-0,04	24,74
O	0,05	-17	46	10	187,31	145,91	106,21	111,31	114,33	107,86	-378,94	-406,59	-375,07	-364,97	-350,04	-341,65	0,27	0,05	36,22
P	0,11	-17	10	10	194,19	139,53	144,93	143,75	140,14	137,40	-372,06	-383,98	-395,47	-400,65	-400,97	-401,29	0,17	-0,04	20,44
Q	0,11	-45	58	2	183,67	139,94	113,92	102,18	97,08	204,62	-347,12	-340,51	-293,12	-280,14	-285,28	-294,97	-0,05	0,08	16,95
R	0,11	-1	58		222,52	146,78	130,85	142,63	114,83	112,21	-363,33	-342,06	-297,52	-354,64	-433,12	-447,56	0,33	-0,10	23,72
S	0,05	-2	38	40	189,11	82,48	129,26	111,64	112,57	114,17	-334,53	-265,35	-334,29	-391,13	-416,16	-425,46	0,25	-0,12	22,60
A3	0,05	-9	62	11	185,63	140,35	40,63	39,12	40,63	46,52	-352,86	-345,28	-213,14	-198,89	-213,14	-207,55	0,60	0,26	208,59
B3	0,05	-9	62	8	217,33	151,12	56,74	52,25	55,01	61,38	-390,47	-385,13	-235,01	-213,49	-211,05	-217,95	0,56	0,28	149,63
C3	0,05	-9	62	10	168,67	142,87	32,05	19,76	17,47	16,23	-256,06	-272,51	-144,89	-112,47	-104,31	-99,94	0,82	0,44	1617,58
D3	0,05	-8	34	8	198,28	175,13	287,35	178,33	174,78	174,28	-366,44	-416,03	-5,90	-393,23	-371,88	-368,20	0,06	0,00	16,55
F3	0,05	-8	34	8	201,19	174,62	181,45	183,09	187,79	194,72	-398,26	-435,30	-444,01	-441,10	-442,07	-449,94	0,02	-0,06	10,89
G3	0,05	-8	36	8	231,59	166,20	171,57	171,73	172,55	171,03	-410,24	-428,15	-410,10	-413,91	-412,78	-416,55	0,15	-0,01	16,02
H3	0,05	-9	62	11	189,57	139,79	42,39	24,21	19,05	17,56	-291,54	-280,65	-162,68	-119,70	-106,16	-100,22	0,83	0,49	1548,05
I3	0,05	-9	62	10	187,89	138,32	35,76	22,50	20,34	19,62	-284,09	-282,27	-149,53	-116,56	-108,42	-105,92	0,81	0,46	1269,76

I3_20	0,05	-9	62	20	206,63	154,96	89,85	53,92	41,12	37,82	-311,55	-317,48	-232,01	-181,38	-160,10	-149,12	0,69	0,35	405,49
J3	0,11	-4	62	11	203,96	156,75	97,15	64,35	55,65	56,91	-302,00	-302,45	-214,41	-160,01	-141,87	-136,19	0,56	0,38	278,07
K3	0,11	-9	62	8	206,53	156,53	99,53	71,77	66,13	68,03	-302,67	-313,84	-217,30	-166,23	-154,12	-151,06	0,50	0,33	195,33
L3	0,11	-4	62	10	200,14	169,70	117,71	94,24	96,73	105,05	-319,17	-332,48	-235,11	-196,71	-201,58	-210,33	0,31	0,21	71,56
N3	0,11	-41	53	24	236,59	196,75	289,41	301,78	257,91	320,41	-372,98	-392,15	-509,77	-518,18	-511,21	-513,87	-0,15	-0,16	4,34
O3	0,11	-8	36	8	153,40	157,01	136,98	128,58	131,66	136,09	-277,37	-283,54	-241,33	-229,03	-226,65	-239,74	0,06	0,07	34,85
P3	0,11	-4	62	8	193,73	129,76	47,27	48,69	59,78	120,02	-267,88	-258,58	-143,47	-143,38	-124,91	-165,09	0,23	0,24	77,12
Q3	0,11	-7	62	11	158,29	105,97	26,13	17,38	12,74	9,93	-256,42	-234,65	-120,23	-100,17	-91,87	-88,31	0,88	0,49	3190,88
R3	0,11	-8	62	10	173,87	112,84	29,08	18,23	13,78	12,29	-281,41	-253,90	-128,21	-102,74	-93,34	-88,46	0,87	0,52	2614,39

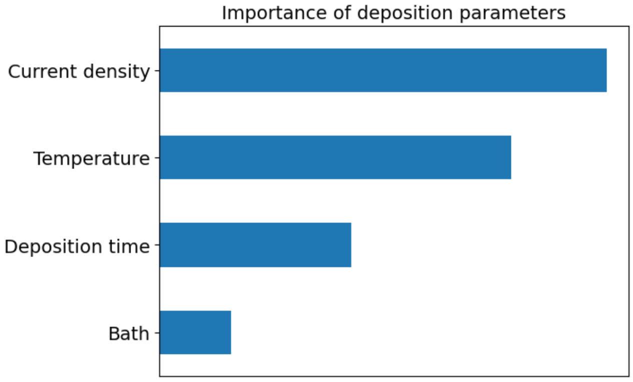

Figure S2. Impurity-based feature importance of the ML model. The importance is proportional to the bar width (arbitrary units). A feature that is more important leads to decision trees splitting the data in a more distinct way.

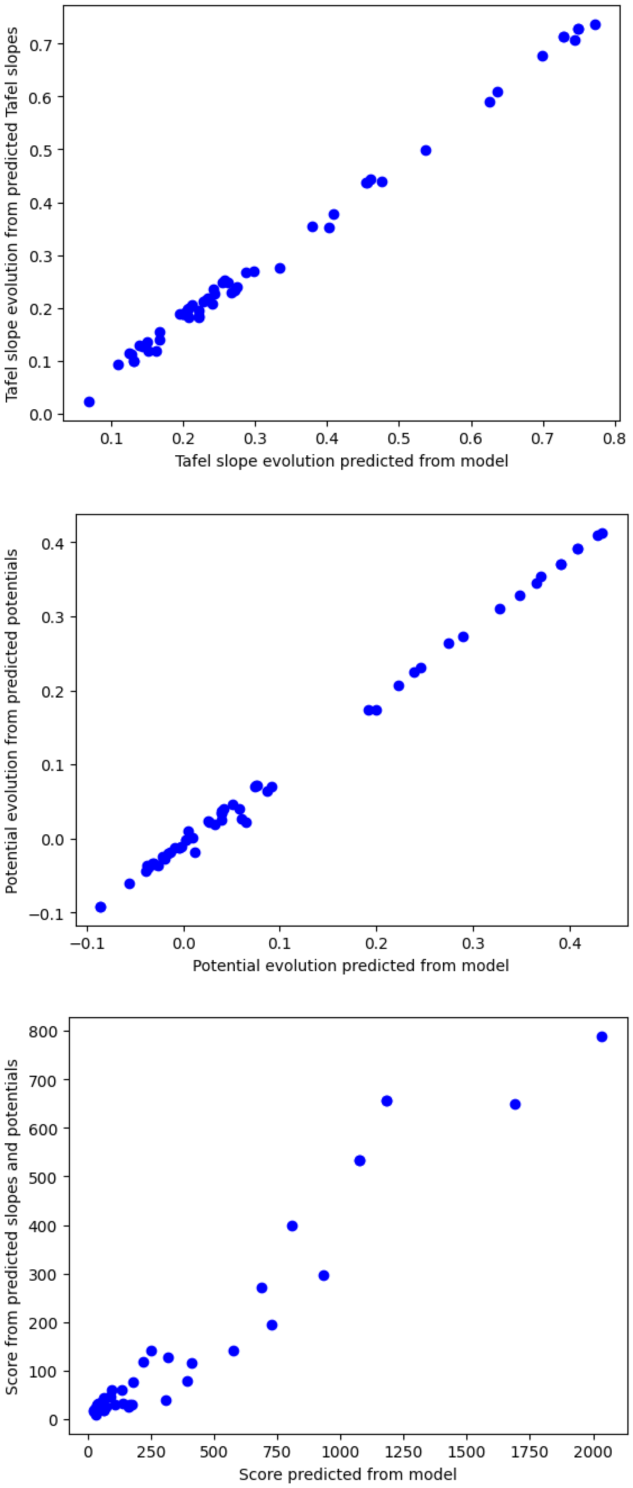

Figure S3. Comparison of the evolution of the Tafel slope (a) and overpotential (b) as predicted directly by the ML model (x axis) and as computed from Eq. (4) and the Tafel slope and overpotential predicted by the ML model (y axis). (c) Comparison of the global score predicted directly by the ML model and computed from Eqs. (4) and (5) and the Tafel slope and overpotential predicted by the ML model.

```
import numpy as np
import pandas as pd

from sklearn.ensemble import RandomForestRegressor


class BaggingRegressorStd(BaggingRegressor):
    """
  Simply extend BaggingRegressor to have an uncertainty on the prediction.
    """

    def predict_std(self, X):
        preds = []
        for estimator in self.estimators:
            preds.append(estimator.predict(X))
        return np.std(preds, axis=0)


# Reading experimental data
# data.csv corresponds to Table S4
df = pd.read_csv("data.csv", index_col=0)

# Getting inputs for the model
X = df[
    [
        "NiSO4 (M)",
        "Na2WO4 (M)",
        "NaGluconate (M)",
        "H3BO3 (M)",
        "P-123 (g)",
        "pH",
        "j (mA/cm^2)",
        "T (ºC)",
        "time (min)"
    ]
].copy()
# Dropping possible duplicate inputs
X = X.T.drop_duplicates().T
# Dropping inputs that are constant over the dataset
X = X.loc[:, (X != X.iloc[0]).any()]
# Filling missing input values by the average over the dataset
X.fillna(X.mean(), inplace=True)

# Getting outputs for the model
y = df[
    [
        "Tafel (mV/dec) 1st cycle",
        "Tafel (mV/dec)
 5th cycle",
        "Tafel (mV/dec) 50th cycle",
        "Tafel (mV/dec) 100th cycle",
        "Tafel (mV/dec) 150th cycle",
        "Tafel (mV/dec) 200th cycle",
        "E (mV) at -10 mA/cm2 1st cycle",
        "E (mV) at -10 mA/cm2 5th cycle",
        "E (mV) at -10 mA/cm2 50th cycle",
        "E (mV) at -10 mA/cm2 100th cycle",
        "E (mV) at -10 mA/cm2 150th cycle",
        "E (mV) at -10 mA/cm2 200th cycle",
        "Potential evolution",
        "Tafel slope evolution",
        "Score"
    ]
].copy()


# Instantiating the model
rf = RandomForestRegressor()
model = BaggingRegressorStd(
    rf,
    n_estimators=50,
    max_samples=0.9
)

# Training the model on the dataset
model.fit(X, y)
```

Figure S4. Representative Python script for training of the machine learning model.
